# The Role of Endoscopy in Investigating the Causes of Persistent Anaemia in Post-operative Fractured Neck of Femur Patients

**DOI:** 10.7759/cureus.47982

**Published:** 2023-10-30

**Authors:** Saurav Krishnan, George Koshy, Anand Reddy, Aysha Rajeev

**Affiliations:** 1 Trauma and Orthopaedics, Gateshead Health Foundation NHS Trust, Gateshead, GBR; 2 Gastroenterology, Gateshead Health Foundation NHS Trust, Gateshead, GBR

**Keywords:** hip fracture, neck of femur fracture, persistent anaemia, gastro-intestinal bleed, endoscopy

## Abstract

Introduction

Post-operative anaemia in hip fracture patients has been associated with increased risk of blood transfusion, poorer functional outcomes, increased morbidity and mortality. Patients with persisting drop in haemoglobin after fractured neck of femur with no obvious source of blood loss are often referred for endoscopy to find the cause of anaemia. The reported incidence of perioperative acute upper gastrointestinal bleeding varies from 1 to 15%.

Objective

The aim of our study is to find out the usefulness of endoscopy in finding gastrointestinal causes leading to the occult loss of blood causing irreversible anaemia in post-operative neck of femur fractures.

Material and methods

The orthogeriatric unit conducted a study using retrospective data on neck of femur fracture patients from January 2015 to December 2020. Out of 1863 cases, 918 (49.3%) developed post-operative anaemia. Forty-five patients (5%) with refractory anaemia underwent endoscopy referral. Patient demographics, fracture patterns, pre-existing anaemia, and co-morbidities (anaemia, heart disease, chronic kidney disease, oral anticoagulant usage) were recorded. The recorded information also included the type of procedure undergone by each patient. Intra-operative tranexamic acid injections were administered to all patients.

Results

Male patients accounted for 24% (11) and females for 76% (34). The average age was 82.3 years (range: 73-94). In terms of fracture type, 60% (27) were intracapsular and 40% (18) were extracapsular. Iron deficiency anaemia was present in 24% (11), oral anticoagulants in 20% (9), and systemic malignancy in 12% (6) of patients. The mean post-operative hemoglobin level during endoscopy referral was 7.3 g/dL. Endoscopy revealed normal findings in 60% (27), esophagitis/gastritis in 20% (8), and hiatus hernia in 16% (7) of patients. No patients were diagnosed with active gastrointestinal bleeding or malignancy as the cause of post-operative hemoglobin drop.

Conclusion

The study did not show evidence of any gastrointestinal bleeding in patients with resistant and refractory post-operative anaemia following fractured neck of femur surgery using endoscopy procedure. The value of such difficult, expensive and time-consuming procedure may be reviewed further.

## Introduction

The surgical treatment of a fractured neck of femur is a widely performed orthopedic procedure globally. This type of surgery is prevalent among the elderly population and is associated with high levels of mortality and morbidity. According to the National Hip Fracture Database (NHFD), approximately 75,000 cases of femoral neck fractures are reported in England and Wales [1]. Among patients undergoing hip fracture surgery, around half of them experience anemia [2], which can be attributed to factors such as frailty, pre-existing medical conditions, and the specific nature of the fracture and surgical procedure. Post-operative anemia in hip fracture patients has been linked to numerous negative outcomes, including an increased likelihood of blood transfusion, reduced functional mobility, and heightened morbidity [2].

Elderly patients with displaced femoral neck fractures are more prone to postoperative anemia, higher American Society of Anesthesiologists (ASA) classification, frailty, and multiple coexisting medical conditions [3]. Both the initial fracture and the surgical intervention can result in blood loss, which can contribute to the development of persistent postoperative anemia.

Anemia is present in approximately 50% of these patients upon admission to the hospital and its incidence increases further following surgery [4]. The mean reduction in the hemoglobin level after surgery is estimated at 0.7-2.5 g/dL [4]. Tranexamic acid [5] has proven to be effective in reducing postoperative anemia, blood loss, the need for transfusion, and blood volume in the drainage. Some of these patients often experience a drop in hemoglobin without an obvious source of blood loss even after blood transfusions and are referred for endoscopy. The reported incidence of perioperative acute upper gastrointestinal bleeding ranges from 0.39% to 14% [6,7]. However, there are currently no established guidelines for managing postoperative anemia in hip fractures and the effectiveness of commonly used methods is uncertain.

The aim of our study is to evaluate the efficacy of endoscopy in identifying any gastrointestinal sources of occult blood loss causing persistent postoperative anemia in patients with a fractured neck of the femur with no obvious gastrointestinal symptoms.

## Materials and methods

Methods

This retrospective, observational study was undertaken in a Queen Elizabeth Hospital, Gateshead, United Kingdom servicing approximately 200,000 people. The audit department of the hospital granted ethical approval for the study. Files of all patients admitted between January 2015 and December 2020 to the orthogeriatric unit for surgical management with fractured neck of the femur were reviewed. Consent to participate was not applicable as this was a retrospective study, only requiring access to patient-level information. The definition of anemia in this study was a hemoglobin (Hb) level of <13 g/dL in men and <12 g/dL in women. Persistent anemia in our study is defined as persisting low levels of Hb below 8 g/dL even after at least two units of blood transfusion.

Data collection

**Figure 1:**
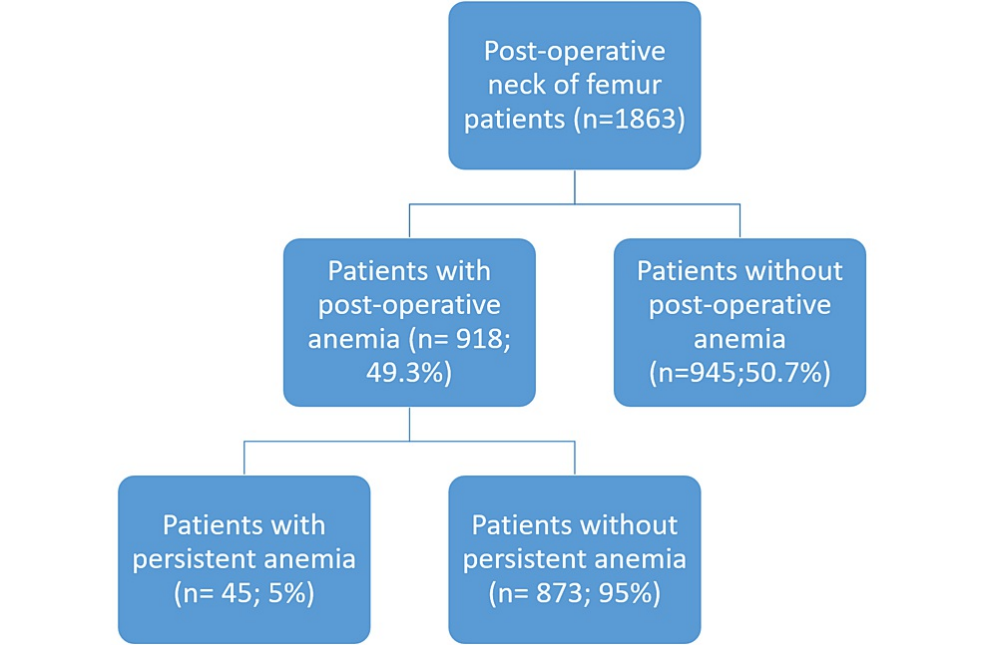
Patients observed from January 2015 and December 2020

Out of 1863 patients admitted with fractured neck of femur, 584 (31.3%) had pre-operative anemia. All these patients were assessed by the multidisciplinary team of elderly medicine, hematologist, and anesthetic team, and the hemoglobin level was optimized with red blood cell transfusion before surgery. Of the 1863 cases, 918 (49.3%) had postoperative anemia. Of these, 45 (5%) patients with persistent anemia underwent an endoscopy to identify any gastrointestinal causes of bleeding contributing to the persistent anemia Figure 1.

The patient demographic data collected included age, gender, fracture pattern, and pre-existing anemia. Other relevant information, such as co-morbidities (anemia, heart disease, chronic kidney disease, and use of oral anticoagulants), and type of surgical procedure was recorded in Table 1.

**Table 1:** Demographic data

Demographics		Number (n;%)
Mean Age		82.3 years(range-73-94)
Sex	Males	11(24%)
	Females	34(76%)
Site of fracture	Intracapsular	27(60%)
	Extracapsular	18(40%)
Medical history		
Iron deficiency anemia		11(24%)
Oral anticoagulants		9(20%)
Systemic malignancy		6(12%)
Peptic ulcer disease		4(8.9%)
Dementia		9(20%)
Atrial fibrillation		11(24%)
Ischemic heart disease		6(12%)
Chronic obstructive pulmonary disease		8(17.8%)
Chronic renal impairment		10(22.2%)
Mean post-op Hb at time of referral		7.7g/dL

All patients received intraoperative tranexamic acid injections. Tabulated data of patients who had non-persistent anemia and were admitted between March 2019 and December 2019 was collected for statistical analysis.

Statistical analysis

The data was checked for completeness and variables for the normality of their distribution. Descriptive statistics for the data are reported as frequencies and percentages. Categorical variables were described using frequencies and percentages and compared between groups using Pearson’s Chi-squared test. P value <0.05 was considered statistically significant. Analyses were performed using the Statistical Package for Social Sciences (SPSS) version 29.0 (IBM Corp., Armonk, NY, USA).

## Results

The study found that the prevalence of postoperative anemia in patients with hip fractures is 49.3%. Forty-five patients were identified with persistent anemia, despite receiving blood transfusions following surgical correction for a fractured neck of the femur. All 45 patients received a minimum of two units of blood postoperatively in an attempt to correct their anemia. The patients had a mean age of 82.3 years, with a range of age distribution from 73 to 94 years. One in three patients with persistent anemia was female. A total of 11 (24%) patients were male and 34 (76%) were female. There were 27 (60%) intracapsular and 18 (40%) extracapsular fractures.

**Figure 2:**
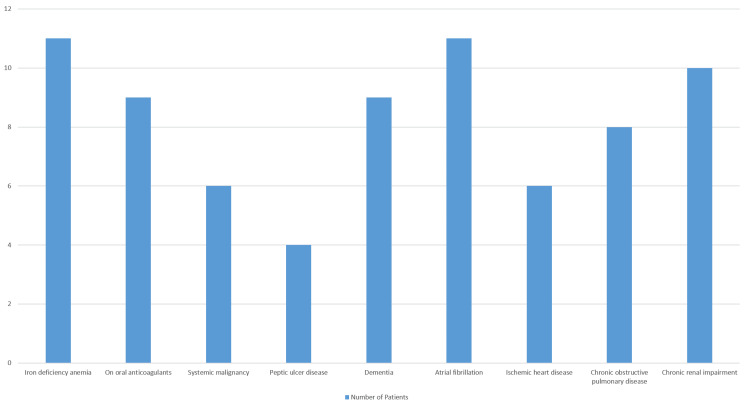
Patients with a diagnosed medical history

Eleven (24%) patients were diagnosed with iron deficiency anemia, nine (20%) were taking oral anticoagulants, and six (12%) had systemic malignancy as in Figure 2.

The mean preoperative hemoglobin was 11.2 g/dL. The mean postoperative hemoglobin level at the time of referral for endoscopy was 7.7 g/dL.

**Figure 3:**
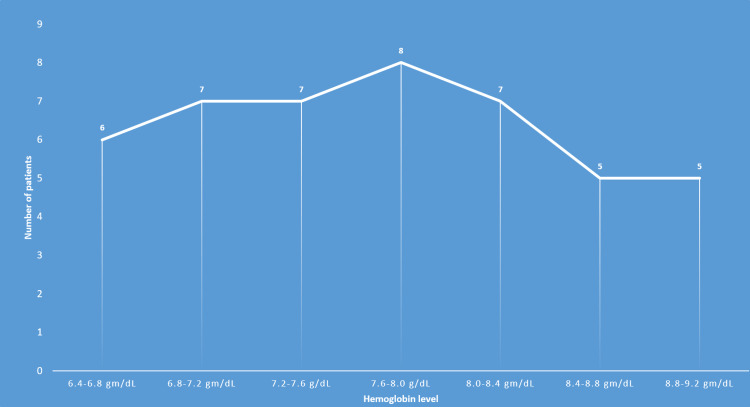
Post-operative hemoglobin levels in gram/deciliter

The number of patients who had upper gastrointestinal endoscopy was 25 (55%), 15 (33%) had colonoscopy and five (12%) had both. The endoscopic evaluation revealed normal findings in 27 (60%) patients, esophagitis/gastritis in eight (20%) patients, and hiatus hernia in seven (16%) patients. None of the patients were found to have an active source of gastrointestinal bleeding or malignancy as the cause of their postoperative decline in hemoglobin as in Figure 3.

The research investigated the factors influencing the persistence of anemia as outlined in Table 2.

**Table 2:** 2 x 2 contingency tables for: A, Type of Fracture; B, Sex; C, Anticoagulation; D, COPD; E, Kidney Disease; F, Systemic Malignancy. Each cell shows the observed cell totals, (the expected cell totals) and {the chi-square statistic for each cell}. The result is significant if p < 0.05. COPD: chronic obstructive pulmonary disease

Demographics	Persistent Anemia	Non-Persistent Anemia	Chi-Square	p-value
Type of Fracture			5.9722	.014533
Intracapsular	18 (25.43) {2.17}	138 (130.57) {0.42}		
Extracapsular	27 (19.57) {2.83}	93 (100.43) {0.55}		
Sex			0.7139	.398159
Males	11 (13.37) {0.42}	71 (68.63) {0.08}		
Females	34 (31.63) {0.18}	160 (162.37) {0.03}		
Anticoagulation			1.0332	.309419
On anticoagulants	9 (11.74) {0.64}	63 (60.26) {0.12}		
Not on Anticoagulants	36 (33.26) {0.23}	168 (170.74) {0.04}		
COPD			0.7779	.377797
COPD	8 (10.27) {0.5}	55 (52.73) {0.1}		
No COPD	37 (34.73) {0.15}	176 (178.27) {0.03}		
Kidney Disease			0.4021	.526008
Kidney Disease	10 (8.48) {0.27}	42 (43.52) {0.05}		
No Kidney Disease	35 (36.52) {0.06}	189 (187.48) {0.01}		
Systemic Malignancy			0.0512	.820935
Systemic Malignancy	6 (5.54) {0.04}	28 (28.46) {0.01}		
No Systemic Malignancy	39 (39.46) {0.01}	203 (202.54) {0}		

Notably our study revealed a noteworthy distinction between extra-capsular fractures and intra-capsular fractures in terms of their impact on persistent versus non-persistent anemia.

## Discussion

The study evaluated a total of 1863 cases, and it was found that 918 (49.3%) of the patients developed post-operative anemia., which is consistent with previous research [4]. This finding highlights the prevalence of anemia among post-operative displaced femoral neck fracture patients and the need for proper monitoring and management of this condition in such patients.

Although the occurrence of anemia in elderly hip fracture patients has been widely documented [2,4], this study further analyzed persistent anemia and endoscopic findings in these patients. The key finding was that none of the endoscopically evaluated patients with persistent anemia showed any signs of active gastrointestinal bleeding or suspected malignancy. This is in contrast to a previous study by Fisher et al., which reported that perioperative acute gastrointestinal hemorrhage occurs in 3.9% of older hip fracture patients [8]. This discrepancy may be attributed to minimal nonsteroidal anti-inflammatory drug (NSAID) use and the widespread use of proton pump Inhibitors in patients with risk factors [9], as most previous research on bleeding in hip fracture patients was conducted prior to this practice. Further investigation is needed to determine the potential impact of quality integrated care, intensive nutritional supplementation, and the selection of deep vein thrombosis (DVT) prophylaxis on the overall outcome. These factors may have also played a significant role and their influence warrants additional research. The statistical analysis has revealed that out of the numerous risk factors assessed, only extracapsular fractures exhibited a notable correlation with an increased risk of persistent anemia. However, further investigation is needed to ascertain the underlying cause behind this association.

The amount of blood lost after surgery for hip fracture, known as total blood loss (TBL), surpasses the blood loss observed during the operation itself [10,11]. The concept of hidden blood loss (HBL) refers to the obvious decrease of hemoglobin during the perioperative period after the fracture, which is inconsistent with the visible blood loss [12]. It has been supported by a plethora of studies that have demonstrated HBL in diverse orthopedic surgical procedures, such as hip fracture surgery [10,11,13], total knee arthroplasty [12,14-16], total hip arthroplasty [17], and spine surgery [18-22]. Pattison et al. suggested that the cause of post-operative blood loss might, in part, be attributed to hemolysis [23]. Research has indicated that hemolysis occurs after the transfusion of a large number of stored red blood cells. Furthermore, general anesthesia has also been associated with hidden blood loss. This is because general anesthesia causes vasodilation in patients, leading to increased intraoperative or post-operative blood loss, as well as increased infiltration of blood into tissue compartments, resulting in a higher amount of hidden blood loss [10].

The limitations of the study include its retrospective design, which prevented the assessment of all possible outcomes and confounding variables such as comorbidities, infection, perioperative complications, and repeated hemoglobin measurements in all patients. Despite the limited sample size, the study provides valuable initial data that supports the need for additional research. Pre-injury hemoglobin values would have been an ideal baseline from which to base further hemoglobin drops. The lack of access to this information is because the study was designed retrospectively and many patients did not regularly have laboratory tests done as outpatients.

Despite the availability of established guidelines for the management of anemia in other surgical patient populations, the management of anemia in geriatric hip fracture patients remains unclear. Additionally, there is a requirement for robust randomized clinical trials to evaluate the role of endoscopy in the management of post-operative anemia in the era of widespread proton pump inhibitor use.

## Conclusions

The presence of anemia in postoperative patients should not be disregarded as it can have significant implications. With the rising prevalence of comorbidities in patients, anemia can become even more complex. However, the efficacy of endoscopy as a management tool for postoperative anemia remains questionable, as evidenced by the findings of our study.
